# Bibliometric analysis and visualization of ferroptosis in metabolic dysfunction-associated steatotic liver disease (MASLD): a comprehensive review

**DOI:** 10.3389/fphar.2026.1856946

**Published:** 2026-08-17

**Authors:** Chensi Yao, Jiahao Sheng, Wei Zheng, Mengjiao Kang, Liyun Duan, Xiao Yuan

**Affiliations:** 1 Department of Endocrinology, The First Affiliated Hospital of Zhejiang Chinese Medical University (Zhejiang Provincial Hospital of Chinese Medicine), Hangzhou, China; 2 The First Clinical Medical School, Zhejiang Chinese Medical University, Hangzhou, China; 3 The First Clinical Medical College, Shandong University of Traditional Chinese Medicine, Jinan, China

**Keywords:** bibliometrics, citespace, ferroptosis, MASLD, NAFLD, trends, vosviewer

## Abstract

**Background:**

Metabolic Dysfunction-Associated Steatotic Liver Disease (MASLD) has emerged as one of the major chronic liver diseases worldwide, and its progression mechanisms have not yet been fully elucidated. Ferroptosis is a form of programmed cell death driven by iron-dependent lipid peroxidation. In recent years, it has been demonstrated to play a crucial regulatory role in the progression of MASLD. Although publications on MASLD have increased rapidly over the past decade, there is currently a lack of systematic bibliometric analysis regarding research trends, emerging hotspots, and global contributions in this field.

**Methods:**

Articles and reviews related to MASLD and ferroptosis were retrieved from the Web of Science Core Collection (WoSCC), PubMed and Scopus. Then duplicate literature and irrelevant literature were removed by Noteexpress software. R software, CiteSpace, VOSviewer and Pajek were employed to analyse and visualise the included literature. Specifically, we examined publication year, country, institution, journal, author, references, and keywords to identify the research landscape and evolutionary trajectory of this field.

**Results:**

This analysis encompassed 373 publications on ferroptosis in MASLD, spanning the period from 2018 to 2026. In the past 4 years, 294 publications were published, accounting for 78.8%. China emerged as the most productive country in this field, contributing 273 publications, with Huazhong University of Science and Technology representing the core research institution. *Frontiers in Pharmacology* was the most influential journal with the highest number of published articles. Wang Hui was identified as the most prolific author. Keyword cluster analysis indicated that lipid peroxidation, iron homeostasis and iron overload constitute current research priorities. Emerging evidence has highlighted the dual roles of iron dysregulation and lipid peroxidation in MASLD pathogenesis, while concurrent efforts have been directed toward bridging traditional Chinese medicine and modern biomedicine in the search for novel therapeutics. Highly-cited literature consistently underscores ferroptosis as a key regulatory node in the progression of MASLD, thereby providing a novel theoretical basis and potential diagnostic-therapeutic targets for this condition.

**Conclusion:**

Research on the association between ferroptosis and MASLD is currently in a phase of rapid development, with research hotspots focusing on the mechanistic exploration of core regulatory pathways and their potential for clinical translation. Future efforts should prioritize strengthening international collaboration and interdisciplinary research. Through large-scale clinical trials and multi-omics data analysis, emphasis should be placed on the clinical translation of ferroptosis-targeted intervention in MASLD treatment. The results of this study may provide an important reference for selecting subsequent research directions in this field.

## 1 Introduction

Non-alcoholic fatty liver disease (NAFLD) is one of the most prevalent chronic liver diseases worldwide, characterized by excessive lipid accumulation in hepatocytes (Rinella et al., 2023). The disease spectrum of NAFLD encompasses non-alcoholic simple fatty liver, non-alcoholic steatohepatitis (NASH), and may progress to liver fibrosis, cirrhosis, and hepatocellular carcinoma. It has become a major public health issue threatening human health. With the deepening understanding of the disease’s pathological mechanisms, the Multi-society Delphi Consensus led by the American Association for the Study of Liver Diseases (AASLD) has standardized the disease nomenclature as metabolic dysfunction-associated steatotic liver disease (MASLD) in 2023 ((Rinella et al., 2024)). This change emphasizes the core feature of “metabolic dysfunction” to better reflect the heterogeneity of patients and the underlying pathophysiology (Méndez-Sánchez et al., 2022).

A meta-analysis revealed that the global prevalence of NAFLD during the study period from 1990 to 2019 was 30.1%, increasing from 25.3% between 1990 and 2006 to 38.2% between 2016 and 2019, representing a 50.4% rise over nearly 3 decades (Younossi et al., 2023). The primary risk factors for MASLD are strongly associated with multiple components of metabolic syndrome, including obesity, diabetes, insulin resistance, and dyslipidemia (Bellentani, 2017). The presence of these risk factors may accelerate disease progression, leading to a sustained rise in MASLD-related mortality (Estes et al., 2018). More importantly, MASLD not only causes local hepatic pathology but also elevates the risk of complications including cardiovascular disease, type 2 diabetes mellitus, chronic kidney disease, and certain extrahepatic cancers, imposing a substantial medical and economic burden on patients and society (Targher et al., 2024). Cardiovascular diseases, extrahepatic malignant tumours and cirrhosis-related complications are ranked as the primary causes of mortality in MASLD patients. However, the pathogenesis of MASLD remains incompletely understood, and targeted therapies are not yet available in clinical practice. Thus, exploring the key regulatory mechanisms underlying disease progression has become a research priority in the field of metabolic disorders.

Ferroptosis, a form of programmed cell death formally named in 2012, is fundamentally characterized by iron-dependent cell death driven by the toxic accumulation of lipid peroxides (Dixon et al., 2012). Cells undergoing ferroptosis exhibit specific morphological changes, including mitochondrial shrinkage, increased membrane density, and reduced or even absent cristae structures. In contrast, the plasma membrane shows a bubbling appearance without significant cellular swelling. This distinct morphological profile serves as a key diagnostic criterion for ferroptosis (Dixon et al., 2012).

Ferroptosis is primarily governed by three core regulatory pathways. An imbalance in iron metabolism induces the Fenton reaction, generating reactive oxygen species (ROS) that provide the substrate for lipid peroxidation. Lipid metabolic dysfunction leads to the synthesis of polyunsaturated fatty acid phospholipids, which are particularly susceptible to ROS attack and subsequent peroxidation. Finally, impaired function of the glutathione peroxidase 4 (GPX4)-centered antioxidant system prevents the timely clearance of lipid peroxidation products, ultimately initiating ferroptosis (Stockwell et al., 2020).

In recent years, a growing body of research has confirmed a close association between ferroptosis and the occurrence and progression of MASLD, providing a novel perspective for elucidating the pathophysiological mechanisms of the disease (Yu and Song, 2024). Beyond aberrant hepatic lipid deposition, patients with MASLD also exhibit markedly elevated systemic and hepatic iron concentrations (Wang H. et al., 2022). The aberrant activation of ferritinophagy mediated by nuclear receptor coactivator 4 (NCOA4) creates a prerequisite for the occurrence of ferroptosis (Ma et al., 2023). Excessive lipid accumulation in hepatocytes of patients with MASLD triggers intense oxidative stress responses, leading to significantly elevated levels of lipid peroxidation products such as malondialdehyde (MDA) and 4-hydroxynonenal (4-HNE) (Miura et al., 2025). Furthermore, the accumulation of 4-HNE protein adducts exacerbates hepatocyte injury and promotes the progression of liver fibrosis (Poli et al., 2008). Further studies have revealed that ferroptosis of hepatic non-parenchymal cells, such as hepatic stellate cells and macrophages, is also involved in the pathological progression of the disease (Xiao et al., 2023; Deng Y. et al., 2024). These findings indicate that ferroptosis is not an isolated cellular event, but a key regulatory link throughout the progression of MASLD from simple fatty liver to metabolic dysfunction-associated steatohepatitis (MASH) and liver fibrosis. It also has an inherent mechanistic association with the core aetiology of “metabolic dysfunction” emphasised in the nomenclature of MASLD.

Bibliometric analysis evaluates publication output, author contributions, and citation impact, providing insights into a field’s progress, challenges, and opportunities. Recent research into metabolic liver diseases and ferroptosis has clarified their regulatory mechanisms, leading to a growing number of studies on their association with MASLD. However, no bibliometric studies focusing on this specific field have been reported to date. This study aims to comprehensively outline the current research status of ferroptosis in MASLD by employing bibliometric and visual analysis techniques. Through a systematic review of the literature, in-depth analyses will be conducted from multiple dimensions, including country, institution, journal, author, reference, and keyword. This will clarify the research trajectory, Frontier hotspots, and development trends in this field, thereby providing directional guidance and conceptual references for exploring ferroptosis-related mechanisms in MASLD and proposing response strategies with the greatest potential for clinical impact.

## 2 Materials and methods

### 2.1 Date sources and retrieval strategy

The data were obtained from the Web of Science Core Collection (WOSCC), Scopus, and PubMed. To prevent fluctuations in search results due to daily database updates, all queries were executed on a single day (2 February 2026). Based on a review of Medical Subject Headings (MeSH) and relevant literature, search strategies were customized for each database. The complete search terms for all databases are presented in Supplementary Tables S1-S3. The search was limited to publications from 1 January 2018 to 2 February 2026, we respectively retrieved 325, 185, and 452 publications from the WOSCC, PubMed, and Scopus databases. With the help of NoteExpress, we ultimately obtained 503 publications after excluding 459 duplicate publications. Then the remaining records were independently screened by two researchers based on titles and abstracts to remove the literature unrelated to the topic (n = 94), meeting abstract (n = 15); editorial material (n = 6); letter (n = 3); early Access (n = 6); correction (n = 5) and retracted publication (n = 1). Through this process, 373 publications were ultimately included for analysis. Meanwhile, all 373 of these publications are in English. The final 373 included articles were exported in NoteExpress software using the export style of web of Science for further analysis.

### 2.2 Analytical methods

**FIGURE 1:**
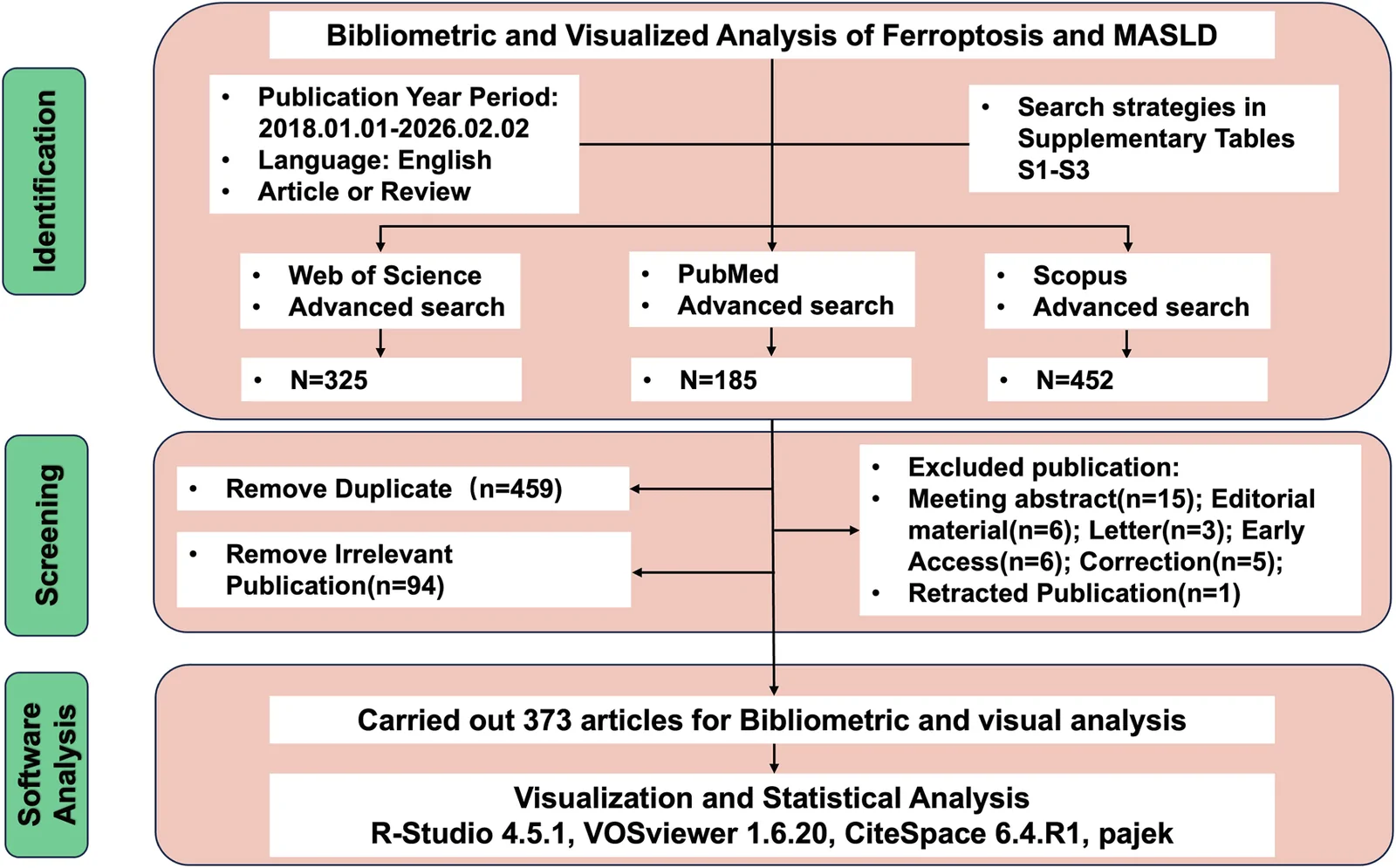
Flowchart of the analysis process.

We applied four tools for bibliometric analysis: VOSviewer (version 1.6.20), CiteSpace (version 6.4. R1), R software (version 4.5.1) and Pajek. VOSviewer was utilized to generate a multidimensional collaborative network (van Eck and Waltman, 2010), visualizing connections among institutions, journals, authors and keywords. Pajek was used to optimize the network layout and enhance visualization. CiteSpace facilitated the exploration of knowledge structures and research trends (Chen and Song, 2019), visualizing connections of countries and producing clustering maps and burst detection maps of keywords. R software, a comprehensive platform for statistical computing and data visualization (Aria and Cuccurullo, 2017), was employed to create a countries’ scientific production map, and Cleveland dot plots. The dot plots highlight the leading contributors in terms of countries, institutions, journals, and authors. The workflow diagram is illustrated in Figure 1.

## 3 Results

### 3.1 Analysis of publication and citation trends

**FIGURE 2:**
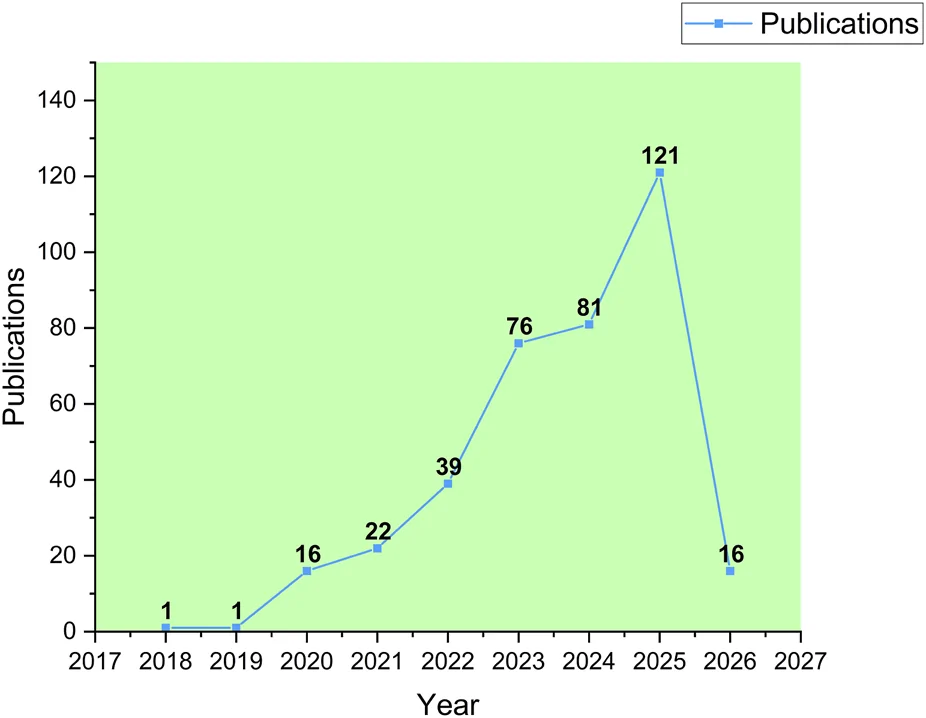
Fitted curves of annual publications frequency for literature related to ferroptosis and MASLD over the last 9 years.

This analysis encompassed 373 publications on ferroptosis in MASLD, spanning the period from 2018 to 2026. As illustrated in Figure 2, annual publication output has exhibited a consistent upward trajectory since 2018. Because the literature search was completed by 2 February 2026, the publication count for 2026 appears lower in the current annual trend graph. This trend indicates that the role of ferroptosis in MASLD has attracted growing research attention and has established itself as an important topic in recent years.

### 3.2 Analysis of countries and institutions

**FIGURE 3:**
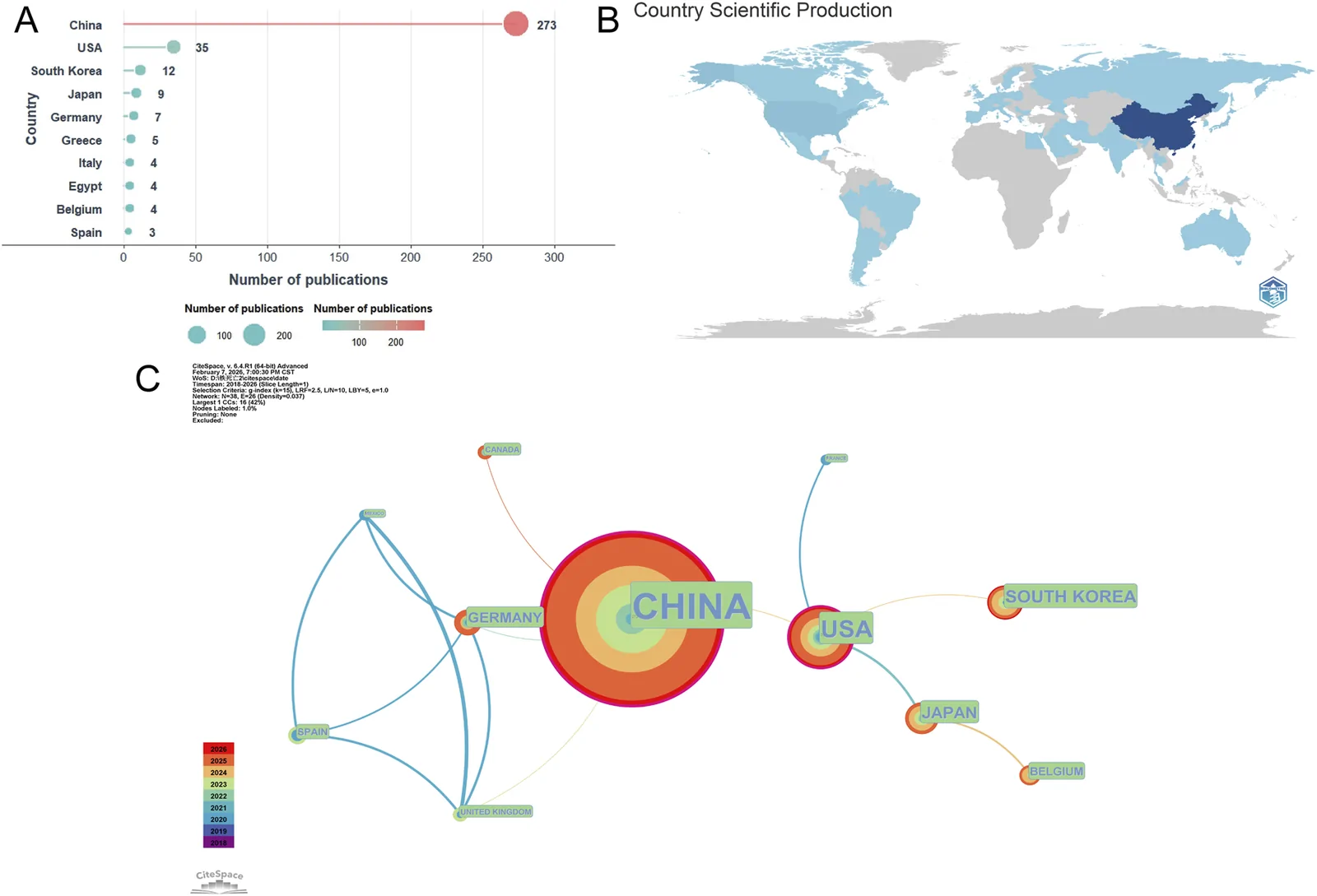
Contribution and collaboration among countries. **(A)** The top 10 most productive countries. **(B)** Contribution among countries. **(C)** Network Visualization Map based on collaboration among countries.

Figure 3A presents the top ten countries by publication volume. In terms of research output, China contributed the most publications (n = 273). Figure 3B maps the global distribution of research activity, further highlighting China’s leading role in this field. The international collaboration network is displayed in Figure 3C, which includes 11 countries. It is evident that China, United States of America, and Germany demonstrate substantial engagement in international collaboration within this research area.

**FIGURE 4:**
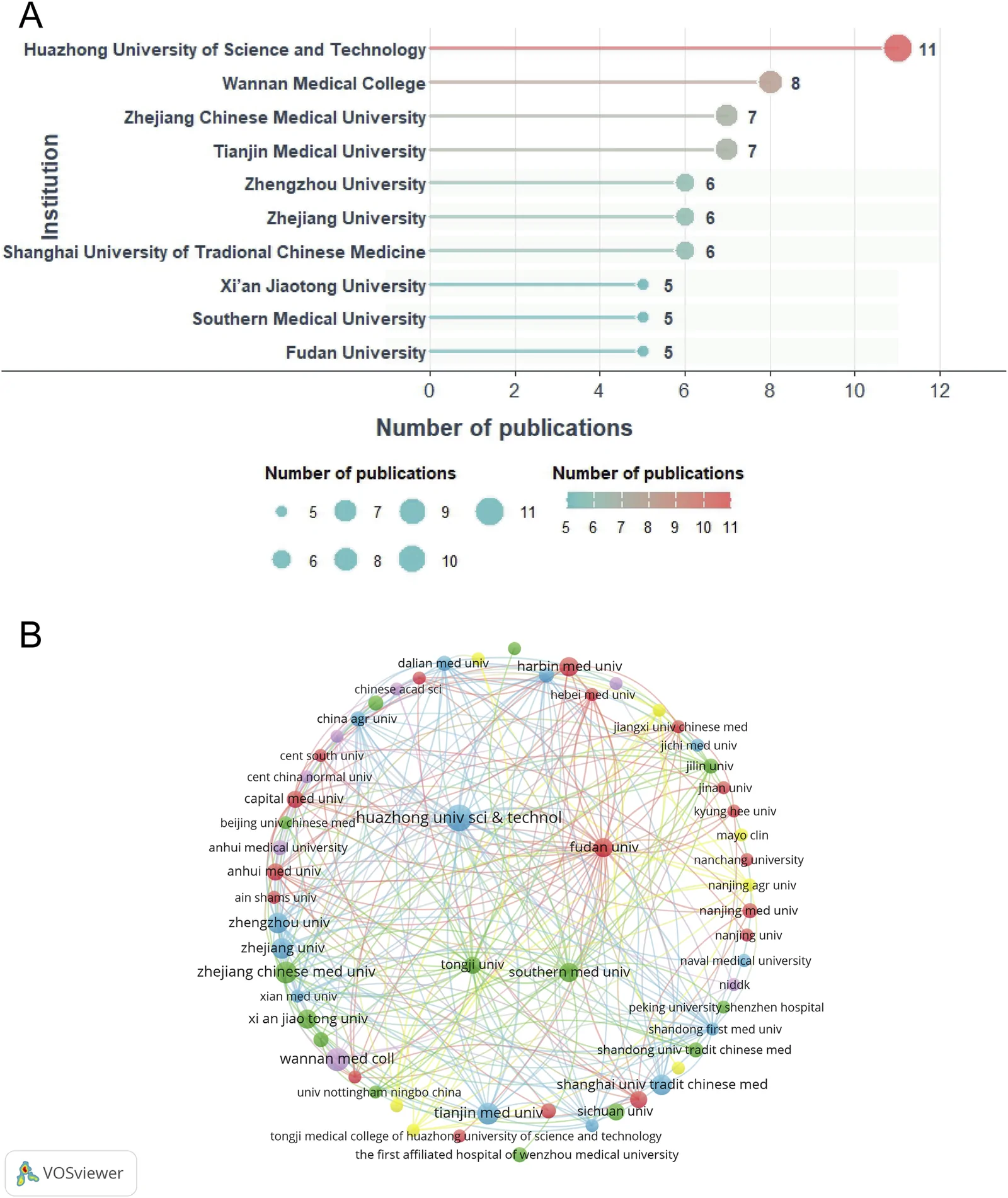
Contribution and collaboration among institutions. **(A)** The top 10 most productive institutions. **(B)** Network Visualization Map with a minimum number of documents of an institution of >2 publications based on collaboration among institutions.

The 373 included publications originated from 376 institutions worldwide. Figure 4A lists the ten most productive institutions in ferroptosis and MASLD research. Notably, all of the top ten are located in China. Huazhong University of Science and Technology led with 11 publications. Huazhong University of Science and Technology published its first article in this field in 2022, and since then, it has published related papers every year. The institutional collaboration network depicted in Figure 4B identifies the most actively collaborating institutions, which include Huazhong University of Science and Technology (60 links), the Fudan University (60 links), Tongji University (46 links), and Southern Medical University (36 links).

### 3.3 Analysis of core journals

**FIGURE 5:**
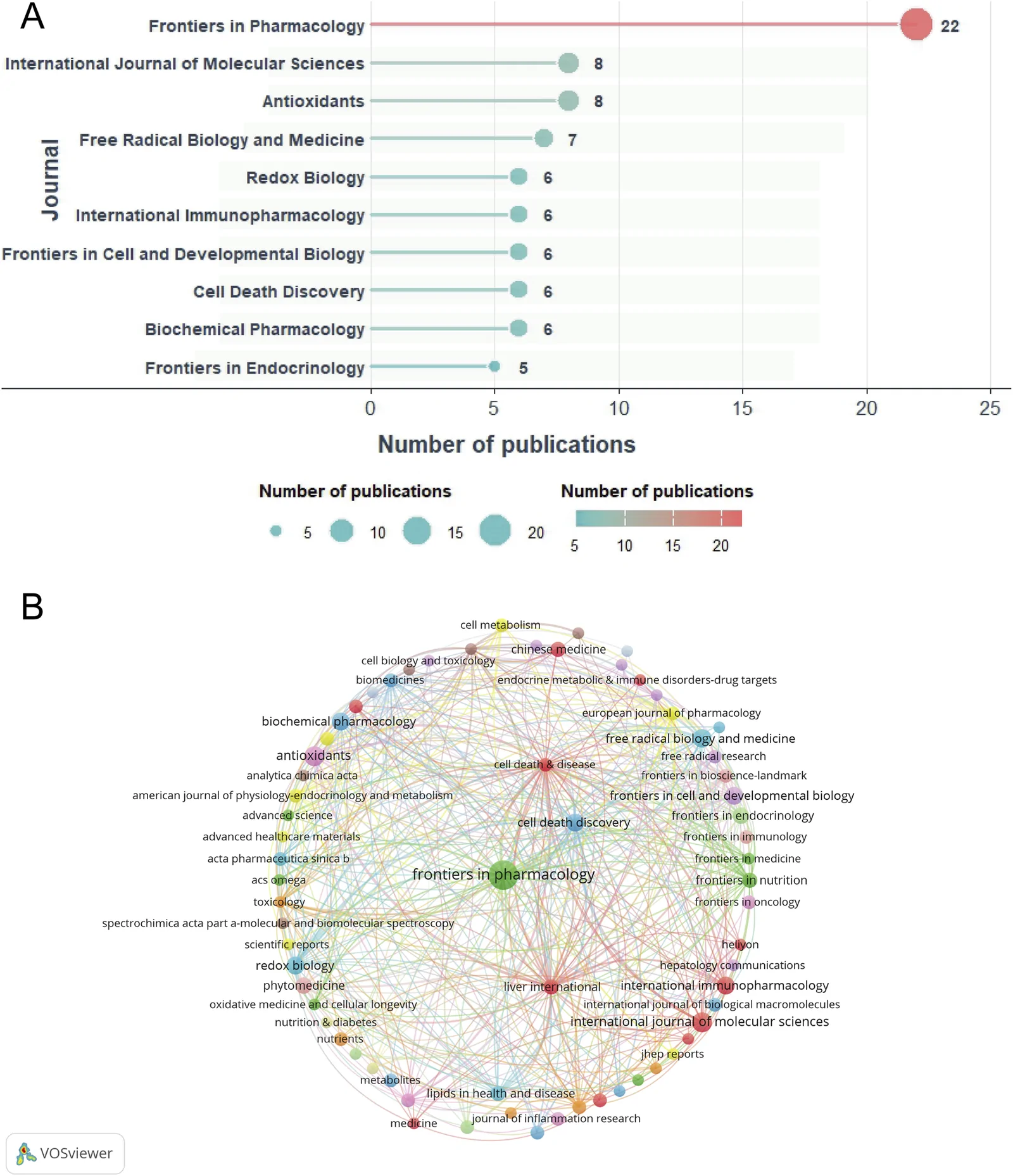
The most productive and influential journals in research on ferroptosis and MASLD. **(A)** The top 10 most productive journals contributing to research. **(B)** Network Visualization Map with a minimum number of documents of a journal of >2 publications.

Figure 5A displays the top 10 journals that published articles on ferroptosis and MASLD between 2018 and 2026. These 10 journals collectively contributed 80 relevant publications, accounting for 21.4% of all included records. Among them, *Frontiers in Pharmacology* (Impact Factor: 4.8, JCR Q1) published the most articles (n = 22), representing 5.9% of the total. Furthermore, the journal network visualized in Figure 5B indicates that *Frontiers in Pharmacology*, *Cell Death and Disease*, *Cell Death Discovery*, and *Liver International* were the core journals with the highest total link strength.

### 3.4 Analysis of authors

**FIGURE 6:**
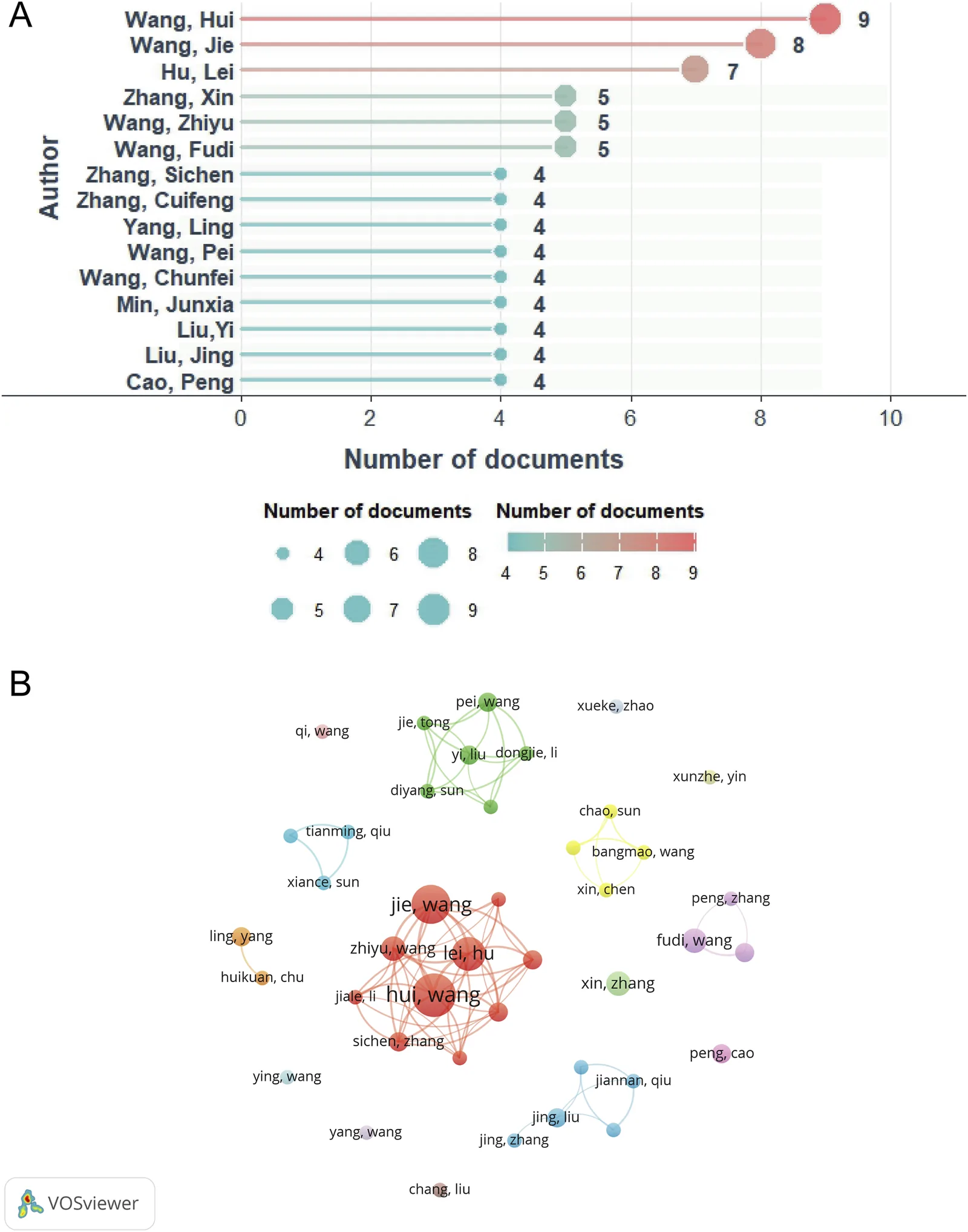
Authors’ contribution and collaboration. **(A)** The top 15 authors’ production over time. **(B)** Network Visualization Map with a minimum number of documents of a author of >3 publications based on collaboration among authors.

As shown in Figure 6A, Wang Hui was the most prolific author in this field, with a cumulative total of nine relevant publications. Wang Hui’s researches mainly focus on 2024 to 2025. Figure 6B displays the collaboration network among authors who have published at least three articles, revealing 15 distinct collaborative clusters. The largest and most interconnected cluster is centered around Wang Hui, whose team has made significant contributions to MASLD diagnosis. Specifically, the team developed lipid droplet fluorescent probes with high polarity sensitivity, such as CNL4 and TST. By monitoring polarity changes in lipid droplets during ferroptosis, these probes enable cross-scale visualization ranging from cellular and tissue levels to whole organs. These advances provide important technical support for the real-time imaging and diagnosis of ferroptosis-associated MASLD (Yang et al., 2024a; Ge et al., 2024; Yang et al., 2024b).

### 3.5 Analysis of citations

**FIGURE 7:**
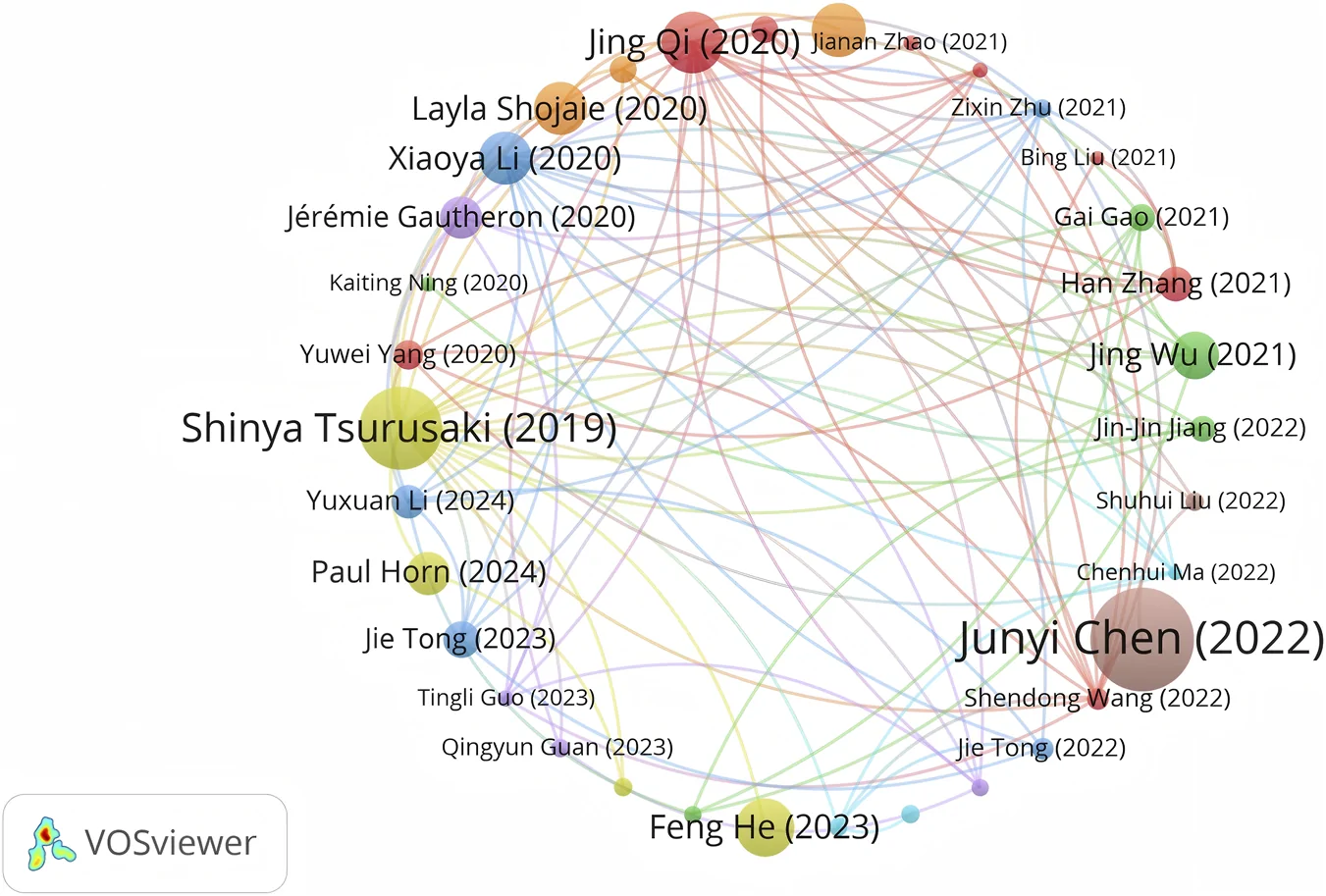
Analysis of citations.

Of the 373 publications included in this analysis, 34 articles have received more than 50 citations. Table 1 lists the ten most cited publications, comprising five research articles and five reviews. In the citation network visualized in Figure 7, node size corresponds to citation frequency, with larger nodes denoting greater scholarly influence. The largest node represents the article by Junyi Chen et al. (Chen et al., 2022) published in *Cell Death and Differentiation*, which ranks first with 529 citations.

**TABLE 1:** The top 10 publications by citations.

Rank	First author	Title	Journal	Year	Citations	JCR	If
1	Junyi Chen	The multifaceted role of ferroptosis in liver disease	*Cell death and Differentiation*	2022	529	Q1	15.4
2	Shinya Tsurusaki	Hepatic ferroptosis plays an important role as the trigger for initiating inflammation in nonalcoholic steatohepatitis	*Cell Death and Disease*	2019	412	Q1	9.6
3	Jing Qi	Ferroptosis Affects the Progression of Nonalcoholic Steatohepatitis via the Modulation of Lipid Peroxidation-Mediated Cell Death in Mice	*American Journal of Pathology*	2020	286	Q1	3.6
4	Feng He	ATF4 suppresses hepatocarcinogenesis by inducing SLC7A11 (xCT) to block stress-related ferroptosis	*Journal of Hepatology*	2023	267	Q1	33.0
5	Xuefei Zhang	Inhibition of tumor propellant glutathione peroxidase 4 induces ferroptosis in cancer cells and enhances anticancer effect of cisplatin	*The Journal of Cellular Physiology*	2020	246	Q1	4.0
6	Layla Shojaie	Cell death in liver diseases: a review	*International Journal of Molecular Sciences*	2020	238	Q1	4.9
7	Xiaoya Li	Targeting ferroptosis alleviates methionine-choline deficient (MCD)-diet induced NASH by suppressing liver lipotoxicity	*Liver International*	2020	236	Q1	5.2
8	Jing Wu	Ferroptosis in liver disease: new insights into disease mechanisms	*Cell Death Discovery*	2021	208	Q1	7.0
9	Paul Horn	Metabolic reprogramming in liver fibrosis	*Cell Metabolism*	2024	188	Q1	30.9
10	Jérémie Gautheron	Lytic cell death in metabolic liver disease	*Journal of Hepatology*	2020	183	Q1	33.0

### 3.6 Analysis of keywords and research hotspots

**FIGURE 8:**
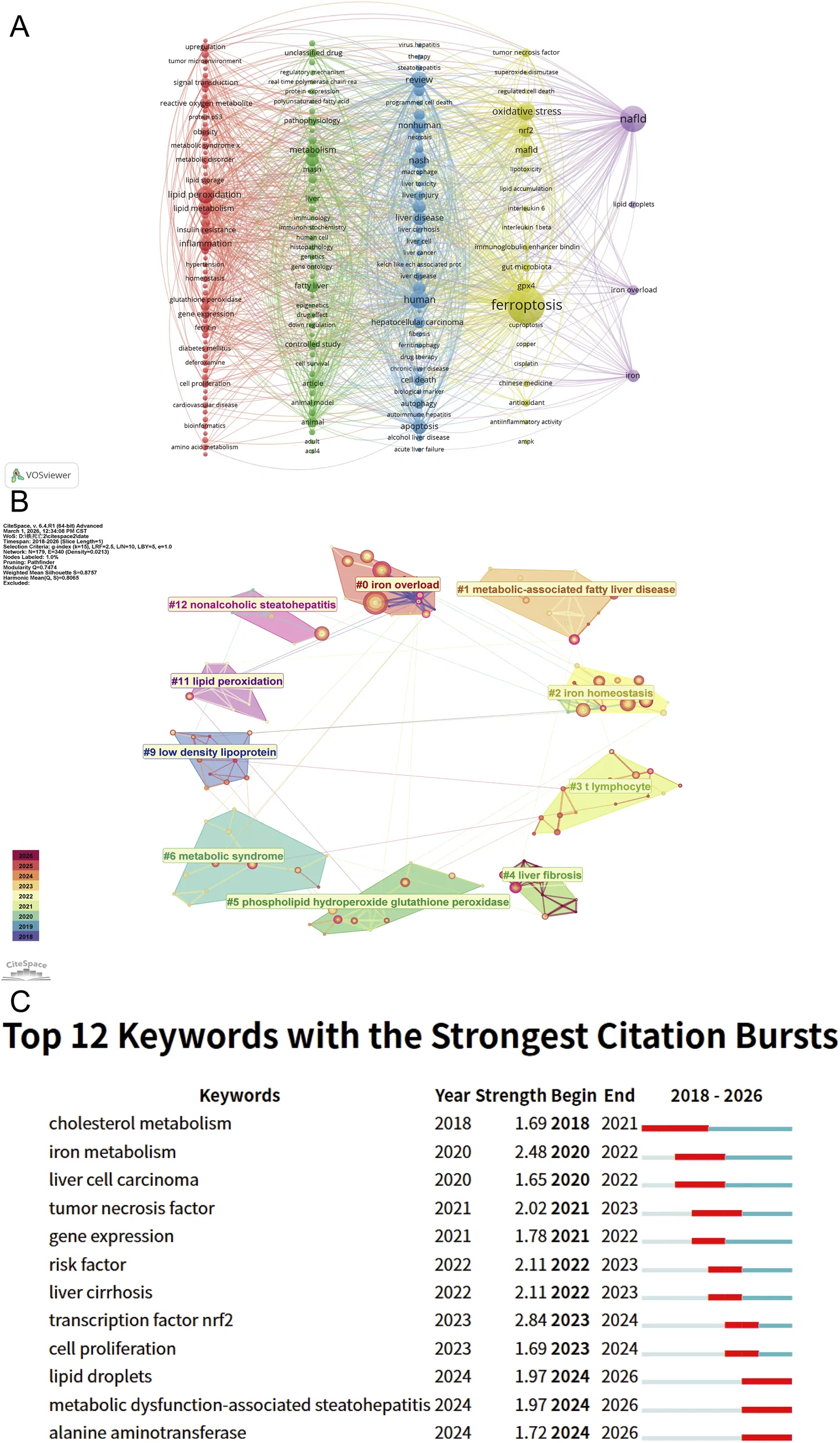
Analysis of keywords. **(A)** Keyword Co-occurrence Network related to ferroptosis and MASLD. **(B)** Keyword Clustering Map between ferroptosis and MASLD. **(C)** Top 12 keywords with the strongest citation burst.

We constructed a keyword co-occurrence network using VOSviewer and optimized the network layout with Pajek to enhance visualization. This analysis identified five distinct clusters (Figure 8A). In the network diagram, each node represents a keyword, with node size reflecting its frequency of occurrence and different colors indicating distinct thematic clusters. Based on the keywords with high co-occurrence frequencies and core associations within each cluster, we categorized the following themes: The red and purple clusters primarily focus on the mechanistic role of ferroptosis in MASLD, encompassing keywords such as “lipid peroxidation”, “lipid metabolism”, and “iron overload”. The green cluster centers on experimental approaches for investigating ferroptosis in this disease, with keywords including “mice”, “animal model”, and “human cells”. The blue cluster pertains to the disease spectrum of MASLD, featuring terms such as “liver disease”, “nash”, “fibrosis”, “liver injury” and “hepatocellular carcinoma”. The yellow cluster addresses the regulation of relevant signaling pathways by ferroptosis, with keywords including “ferroptosis”, “Nrf2”, “GPX4”, “AMPK” and “interleukin 6”.

A keyword cluster analysis was performed using CiteSpace, generating the clustering network presented in Figure 8B. The CiteSpace parameters were configured with a time slicing of 1 year, a Top N per slice value of 15, and pathfinder network scaling combined with pruning of sliced networks and pruning of the merged network for network simplification. The latent semantic indexing (LSI) algorithm was employed for keyword clustering. The network demonstrated a well-defined structure with significant clustering validity (modularity Q = 0.7337; mean silhouette S = 0.9298). The research themes represented by each cluster are summarized as follows: Clusters #0 (‘iron overload’),#2 (‘iron homeostasis’),#11 (‘lipid peroxidation’), and #5 (‘phospholipid hydroperoxide glutathione peroxidase’) are closely associated with the mechanistic foundations of ferroptosis (Ru et al., 2024; Zhang W. et al., 2024; Zheng and Conrad, 2020). Clusters#4 (‘liver fibrosis’) and #12 (‘nonalcoholic steatohepatitis’) correspond to key pathological stages within the disease spectrum of MASLD. Cluster#1 (‘metabolic-associated fatty liver disease’) reflects the evolving nomenclature of this condition. Cluster #3 (‘t lymphocyte’) suggests a potential regulatory role of T lymphocytes in ferroptosis (Liao et al., 2022). Cluster #6 (‘metabolic syndrome’) highlights the contributory role of metabolic syndrome in the initiation and progression of MASLD (Muzurović et al., 2021). Cluster #9 (‘low density lipoprotein’) indicates that abnormally elevated low-density lipoprotein levels during disease progression may accelerate liver fibrosis and cirrhosis, while concurrently increasing cardiovascular disease risk (Deprince et al., 2020).

Figure 8C presents the keyword burst map. We Set the detection model’s γ to 0.7 and keep the other parameters at their default values. “Transcription factor Nrf2” exhibited the highest burst strength (strength = 2.84), with its burst period spanning from 2023 to 2024, indicating that Nrf2—as a key signaling pathway in ferroptosis (Tang et al., 2021)—has been a research focus in recent years. Keywords such as “lipid droplets”, “metabolic dysfunction-associated steatohepatitis” and “alanine aminotransferase” showed burst periods concentrated between 2024 and 2026, suggesting that these topics may represent emerging research directions.

### 3.7 Pharmacological intervention landscape identified in the literature

Beyond the thematic clusters identified above, we further examined the pharmacological intervention studies within the 373 analyzed publications. These studies constituted a substantial proportion of the included literature, with natural phytochemicals and traditional Chinese medicine (TCM) formulas representing a major category, alongside synthetic compounds, Western medicines, and nanoparticle-based delivery systems. Notably, the Nrf2-GPX4 antioxidant axis was the most frequently targeted pathway across these intervention studies—a finding that directly aligns with its strongest keyword burst (Figure 8C). Other commonly modulated mechanisms included iron chelation (corresponding to Cluster #0, ‘iron overload’), inhibition of lipid peroxidation (Cluster #11), and regulation of gut microbiota, all of which are consistent with the dominant mechanistic clusters identified in our keyword co-occurrence network. Detailed mechanisms of representative agents are summarized in Table 2 and 3 in Section 4.4.

**TABLE 2:** Phytochemicals and Chinese herbal medicine therapeutic strategies.

Medicine	Mechanism/Pathway	Model	References
Ginkgolide B (GB)	increase Nrf2	HFD-induced mice HepG2 cells	Yang et al. (2020)
Dehydroabietic acid (DA)	activate Nrf2-ARE	HFD-induced mice	Gao et al. (2021)
Icariin	activate Nrf2/SLC7A11/GPX4 axis	HFD-induced mice	Choi et al. (2023)
Dehulled adlay	activate AMPK/Nrf2 pathway and inhibit NLRP3 inflammasome; modulate gut microbiota	HFD-induced rats	Huang et al. (2023)
Arbutin (ARB)	inhibit FTO upregulate SLC7A11	HFD-induced mice	Jiang et al. (2023a)
Atractylodin (ART)	activate NFE2L2/NRF2 pathway, upregulate GPX4, FTH1, and SLC7A11	HFD-induced mice HepG2 cells	Ye et al. (2023)
Berberine	activate SLC7A11/GSH/GPX4	HFD-induced mice	Gu et al. (2024)
Atractylodes lancea rhizome (AP)	suppress KEAP1/NCOA4 activate NRF2-FTH1/GPX4	MCD-induced mice	Pi et al. (2024)
Nuciferine (Nuc)	activate PPARα	HFD-induced mice	Qiu et al. (2024b)
Lycorine	activate EGFR/PI3K/AKT	HFD-induced mice	Wang et al. (2024c)
Echinacoside (ECH)	activate Nrf2/HO-1	db/db mice HepG2 cells	Yan et al. (2024b)
Diosgenin	activate Nrf2 pathway, activate FSP1/CoQ10 axis, inhibit ACSL4/LPCAT3/ALOX15	HFD-induced rats Human liver carcinoma cells HepG2 cells	Zhang et al. (2024b), Wang et al. (2025a)
Salvianolic acid A (SAA)	activate AMPK/IGFBP-1	Western diet-induced mice	Zhu et al. (2024)
Camellia japonica radix (CJR)	regulate gut microbiota regulate linoleic acid metabolism upregulate GPX4	HFD-induced mice AML12 cells	Gu et al. (2025b)
Oroxylin A	activate Keap1-Nrf2-GPX4-SLC7A11 upregulate Nrf2/HO-1	HFD-induced mice	Jiang et al. (2025)
(−)-Epicatechin	suppress NF-κB and activate Nrf2 pathway	HFD-induced mice, hepG2 cells	Li et al. (2025)
Tricholoma mongolicum polysaccharide (TMP)	activate Nrf2-GPX4/FPN1, suppress TFR1	HFD-induced mice	Ma et al. (2025)
Fucoxanthin (Fx)	suppress hydroxyproline/α-SMA, upregulate Nrf2/HO-1/NQO1/GCLM/GPX4/SLC7A11/TFR1/FTL	CCl_4_ injection-induced liver fibrosis	Liu et al. (2025b)
Pachymic acid (Pac)	suppress MAPK activate PPARα	HFD-induced mice	Ren et al. (2025)
Hinokitiol (Hino)	activate Nrf2	HFHC-induced mice, PO-stimulated hepatocytes	Yin et al. (2025)
Tectorigenin (TEC)	activate HDAC1	human hepatocellular carcinoma cell line HepG2	Zhu et al. (2025)
Epigallocatechin gallate (EGCG)	modulate gut microbiota	HFD-induced mice	Ning et al. (2020)
Zeaxanthin (ZEA)	suppress p53	FFA-induced HepG2 cells	Liu et al. (2023)
protocatechuic acid (PCA)	activate Nrf2	both NRF2^+/+^ and NRF2^−/−^ cell lines, PA and HFD- induced mice	Feng et al. (2024)
Erchen decoction (ECD)	activate caveolin-1 (CAV-1)	(PA + OA)-induced hepatocyte HFD-induced mice Cav-1 knockdown cell lines	Deng et al. (2024b)
Chaihu Shugan powder (CSP)	activate AMPK/mTOR/autophagy axis and Nrf2/KEAP1/GPX4 pathway	MCD-induced mice	Liang et al. (2024)
Hu Gan Tang (HGT)	activate Nrf2- GPX4	HFD-induced rats HepG2 cells	Cui et al. (2025)
Yunnan medicine Jiangzhi ointment (YMJO)	activate AMPK	HFD-induced rats BRL 3 A cells	Hong et al. (2025)
Lipi Jiangzhuo decoction (LPJZD)	activate PINK1/GPX4 axis, suppress PERK signaling	HFD-induced mice with fatigue protocols and cold stress	Pan et al. (2025)
Lang Qing A Ta (Huagan Tongluo Fang, HGTLF)	activate GPX4 expression	CCl_4_ injection-induced liver fibrosis HFD-induced mice	Qiu et al. (2025)
Shugan Xiaozhi (SGXZ)	regulate p53/SLC7A11/GPX4	MCD-induced mice	Wang et al. (2025b)

**TABLE 3:** Modern biomedical approaches.

Drug	Mechanism/Pathway	Model	References
Thymosin beta 4 (Tβ4)	upregulate GPX4	HFD-induced rats	Zhu et al. (2021b)
Bone morphogenetic protein 4 (BMP4)	activate GPX4	HFD and high-fructose diet-fed mice, HepG2 and LO2 cells	Wang et al. (2022b)
Oleic acid	activate S6K1	human hepatocytes, immortalized mouse hepatocyte cells	Pardo et al. (2015)
S-nitroso-N-acetylcysteine (NAC-SNO)	activate Nrf2/HO-1	AML12 cells	Abu-Halaka et al. (2023)
Vitamin E (vit E)	activate Nrf2	C57BL/6 male mice	Baratz et al. (2023)
Melatonin (Mel)	inhibit MT2/cAMP/PKA/IRE1 axis; activate Nrf2/HO-1/GPX4/SLC7A11 pathway	HFD-induced mice, hepG2 cells	Gu et al. (2023), Saha et al. (2023)
Liraglutide	activate AMPK/ACC	HFD-induced mice with injection of streptozotocin	Guo et al. (2023)
Cilostazol	prevents ectopic erythrophagocytosis	NASH mouse model named STAM™	Park et al. (2023)
Ferroptosis inhibitor liproxstatin-1 (LPT1)	inhibit ferroptosis (with potential PANoptosis involvement	HFD-induced mice with 30% fructose in water	Tong et al. (2023)
Iron chelator deferiprone (DFP)	inhibit ACSL4/ALOX15	HFD-induced mice with 30% fructose in water	Tong et al. (2023)
Metformin	activate AMPK	HFD-induced rats PA-treated WRL68 cells	Yue et al. (2023)
Hepatocyte-targeted hydrogen delivery system (MSN-Glu) by modifying magnesium silicide nanosheets (MSN) with N-(3-triethoxysilylpropyl) gluconamide	inhibit Fenton-like reaction	HFD-induced mice AML12 hepatocytes	Zhao et al. (2023)
a PEGylated thiolated hollow mesoporous silica nanoparticles (MSN) loaded with OCA, as well as a ferroptosis inhibitor liproxsatin-1 and a NO donor S-nitrosothiol (ONL@MSN)	activate FXR, inhibit ferroptosis and fibrosis	MCD-induced mice AML12 cell line	Fu et al. (2024)
Apomorphine	activate Nrf2	hepatocyte-specific PTEN KO mice Huh 7 and primary cultured hepatocytes isolated from the mice	Maeda et al. (2024)
FerroTerminator1 (FOT1)	inhibit hepatic iron overload, suppress c-Myc/ACSL4 axis	MCD-induced mice HFHC-induced mice, (WD + CCl4)- induced mice	Tao et al. (2024)
Palmitoleic acid (PA)	inhibit ACSL4, activate GPX4/SLC7A11	HFD-induced mice	Wang et al. (2024a)
a liver targeted 18-beta-Glycyrrhetinic-acid-chitosan oligosaccharide -N-acetylcysteine polymer (GCNp), and further curcumin (Cur) was used as model drug to prepare Cur loaded nanodelivery system (GCNp-Cur NPs)	activate GPX4/GSH	HepG2 cells,ICR mice	Yu et al. (2024)
Coenzyme Q (CoQ) and selenium (Se)	activate GPX4	MCS or MCD diet- induced mice	Choi et al. (2025)
novel selenium nanoparticles (SeNPs) are synthesized using *Lactobacillus* coryniformis ES23	activate PPARα	HFD-induced mice	Zhang et al. (2025b)
Ghrelin	activate ASCT2/mTORC1/SCD1 axis; inhibit IRE1α/XBP-1 pathway	HFD-induced mice HepG2 cells	Zhao et al. (2025)

## 4 Discussion

### 4.1 General information

In the following discussion, we structure our synthesis according to the bibliometric findings presented in Section 3. The keyword co-occurrence network (Figure 8A) and cluster analysis (Figure 8B) identified five major thematic clusters, among which Clusters #0 (‘iron overload’), #2 (‘iron homeostasis’), and #11 (‘lipid peroxidation’) emerged as the most persistent mechanistic focus. Meanwhile, burst detection (Figure 8C) highlighted ‘transcription factor Nrf2’ as the term with the highest burst strength, indicating that antioxidant pathway regulation has been a recent research priority. Guided by these quantitative mappings, we systematically review the core regulatory modules underlying ferroptosis in MASLD, with particular emphasis on the pathways reflected by the highly cited references listed in Table 1.

This study analyzed literature on ferroptosis and MASLD over the past 9 years. The results showed that the number of publications increased annually from 2019 to 2025, indicating growing academic attention to research on ferroptosis and MASLD. Among countries, China contributed the most publications and was the most prominent contributor. Besides that, the US has advantages in the molecular mechanisms of ferroptosis and clinical translation, focusing on functional validation of key regulators like ACSL4 (Chen et al., 2023). Japan has made important contributions regarding the timing relationship between inflammation initiation and ferroptosis (Tsurusaki et al., 2019a). Among institutions, Huazhong University of Science and Technology published the most articles and played a key role in this field. *Frontiers in Pharmacology* was the most influential journal, with the highest number of publications and extensive collaborative relationships. Among authors, Wang, Hui was the most prominent scholar, with the highest number of publications and the largest collaborative team. They have made significant contributions to the diagnosis of MASLD.

### 4.2 Key regulatory mechanisms underlying ferroptosis in MASLD

**FIGURE 9:**
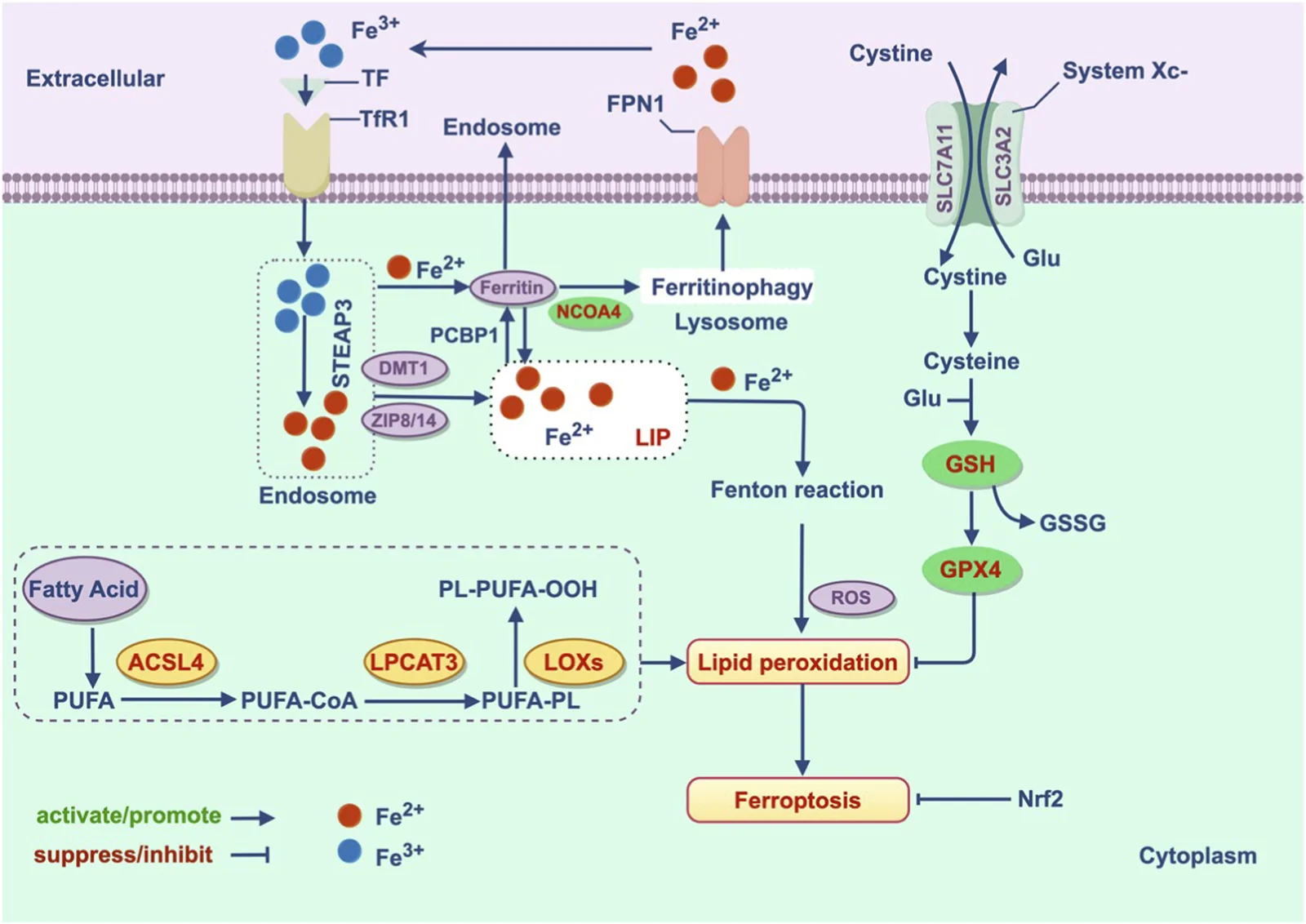
Key Mechanisms of Ferroptosis in MASLD. Dysregulated iron metabolism in the liver perturbs the redox balance of hepatocytes during MASLD development, thereby inducing ferroptosis, promoting the accumulation of lipid reactive oxygen species, and exacerbating the progression of MASH. Abbreviations: TF, transferrin; TfR1, transferrin receptor 1; FPN 1, ferroportin 1; SLC3A2, solute carrier family three member 2; SLC7A11, solute carrier family seven member 11; STEAP3, six transmembrane epithelial antigen of prostate 3; Glu, glutamate; DMT1, divalent metal transporter 1; ZIP8/14, zinc/iron regulatory protein family 8/14; PCBP1, poly (rC)-binding protein 1; NCOA4, nuclear receptor coactivator 4; LIP, labile iron pool; GSH, glutathione; GSSG, oxidized glutathione; GPX4, glutathione peroxidase 4; ROS, oxygen species; Nrf2, nuclear factor erythroid 2-related factor 2; PUFA, polyunsaturated fatty acid; ACSL4, long-chain acyl-CoA synthetase family 4; PUFA-CoA, polyunsaturated fatty acyl CoA; LPCAT3, lysophosphatidylcholine acyltransferase 3; PUFA-PL, polyunsaturated fatty acid-containing phospholipid; PL-PUFA-OOH, Phospholipid-polyunsaturated fatty acid hydroperoxide; LOXs, lipoxygenase.

Ferroptosis is an iron-dependent, lipid peroxidation-driven form of programmed cell death that represents a key pathological driver in the progression of MASLD from simple steatosis to MASH, liver fibrosis, and HCC. We categorize its physiological and pathological mechanisms in MASLD into three interrelated core modules, as illustrated in Figure 9, and summarize the supporting research evidence.

#### 4.2.1 Iron homeostasis dysregulation

Consistent with the identification of Clusters #0 (‘iron overload’) and #2 (‘iron homeostasis’) in our keyword clustering analysis (Figure 8B), and the sustained burst of ‘iron metabolism’ as a research front (Figure 8C), dysregulated iron metabolism emerged as the most frequently discussed mechanistic driver in the analyzed literature. The liver serves as the central hub for systemic iron metabolism, regulating iron storage, transport, and homeostasis via the secretion of key mediators. In addition to serving as a protein-bound element involved in essential cellular processes (including ATP production, DNA synthesis and repair, and oxygen transport), iron also acts as an indispensable component of the redox system. It exerts multiple crucial functions through valence state conversion between ferrous iron (Fe^2+^) and ferric iron (Fe^3+^) (Galy et al., 2024). Clinically, elevated serum ferritin—a surrogate marker of hepatic iron stores—is observed in approximately one in four (26.36%) patients with MASLD, with higher prevalence in biopsy-proven cases (32.61%) and Asian populations (30.89%), and has been consistently associated with more severe histological features, including steatohepatitis and significant fibrosis (Kowdley et al., 2012; Souza et al., 2026). A large population-based study of 5,927 participants confirmed a positive correlation between serum ferritin levels and both steatosis and fibrosis severity (Li et al., 2024). Among patients diagnosed with type 2 diabetes, elevated serum ferritin correctly identified around 80% of those concurrently affected by MASLD; over one-third of this subgroup also presented with substantial liver fibrosis (Amangurbanova et al., 2025). Additionally, serum ferritin predicts long-term prognosis in individuals with MASLD, further validating its value as a non-invasive marker to stratify disease severity and track disease progression (Armandi et al., 2024). These clinical observations substantiate the pathogenic relevance of iron-driven ferroptosis in MASLD progression from simple steatosis to MASH and fibrosis.

Under physiological conditions, most Fe^3+^ is bound to transferrin (TRF) for systemic delivery and stable storage, which buffers fluctuations in circulating iron levels. A smaller fraction enters the labile iron pool (LIP) to meet essential metabolic demands. Transferrin-bound iron (TBI) in the circulation binds to the transferrin receptor 1 (TfR1) on hepatocyte surfaces, which mediates its cellular uptake via endocytosis (Gao et al., 2015). Within the intracellular lysosomes, Fe^3+^ can be reduced to Fe^2+^ by six transmembrane epithelial antigens of prostate 3 (STEAP3). Subsequently, either the zinc-iron regulatory protein family 8/14 (ZIP8/14) or divalent metal transporter 1 (DMT1) mediates the release of Fe^2+^ into the cytoplasm, thereby forming the LIP (Bogdan et al., 2016). Free Fe^2+^ is delivered to ferritin by poly (rC)-binding protein 1 (PCBP1). The ferritin heavy chain 1 (FTH1) oxidizes Fe^2+^ to Fe^3+^, maintaining intracellular iron homeostasis. A separate pool of free Fe^2+^ can also be transported into mitochondria to support physiological functions (Protchenko et al., 2021). Alternatively, intracellular iron can be exported to the extracellular space via ferroportin (FPN). Hepcidin secreted by the liver is a regulatory hormone primarily targeting FPN (Das et al., 2020). By inducing the endocytosis and degradation of FPN, it inhibits iron export into the systemic circulation and maintains systemic iron homeostasis (Nemeth et al., 2004). Additionally, a portion of Fe^2+^ can directly enter cells via solute carrier family 39 member 14 (SLC39A14) and be further released into the LIP (Jenkitkasemwong et al., 2015). HIF-2α, which is regulated by iron and oxygen, can mediate the absorption and transport of intestinal iron by directly targeting multiple key intestinal iron transporters, including FPN, DMT1, and DCb (Shah et al., 2009). Overexpression of HIF-2α in the liver promotes extracellular iron accumulation and suppresses the expression of GSH and GPX4. Both hepatocyte-specific HIF-2α knockout experiments and deferoxamine (DFO) intervention can effectively ameliorate this phenomenon, indicating that HIF-2α-mediated iron metabolic disorders play a crucial role in the maintenance of lipid homeostasis and the regulation of ferroptosis (Yan R. et al., 2024).

Therefore, enhanced ferritin breakdown, elevated TfR1 expression, or reduced FPN1 expression is sufficient to drive abnormal expansion of the intracellular LIP. Excess Fe^2+^ within this pool triggers the Fenton reaction, producing highly reactive oxygen species that promote excessive ROS release and exacerbate lipid peroxidation (Haschka et al., 2021). Meanwhile, iron is an essential cofactor required for lipoxygenase (LOXs) activity. By facilitating LOXs-mediated oxidation of polyunsaturated fatty acids, it promotes extensive lipid peroxide accumulation, which in turn triggers ferroptosis (Xie et al., 2022). Ferritin, NCOA4, and TfR1 collectively regulate iron uptake, storage, and degradation. These molecules may therefore serve as biomarkers for the severity of iron dysregulation and the risk of ferroptosis in MASLD (Zhu et al., 2023). Notably, hepatocytes can release excess intracellular iron to hepatic stellate cells via an extracellular vesicle (EV)-mediated iron transport mechanism, inducing iron accumulation in these cells and further promoting the progression of hepatic fibrosis (Gao et al., 2022).

#### 4.2.2 Lipid peroxidation imbalance

The prominence of ‘lipid peroxidation’ as an independent cluster (#11) in our CiteSpace analysis (Figure 8B), together with its high co-occurrence frequency with ‘oxidative stress’ in the VOSviewer network (Figure 8A), corroborates its critical role as the executioner of ferroptosis in MASLD. In addition to iron overload, ferroptosis is characterized by lipid peroxidation through both enzymatic and non-enzymatic pathways. This process ultimately results in the accumulation of toxic peroxidation products and subsequent cell death (Qiu B. et al., 2024). Research indicates that over 90% of MASLD patients exhibit elevated concentrations of the lipid peroxidation products MDA and 4-HNE (Loguercio et al., 2001). The accumulation of 4-HNE protein adducts in liver biopsies has been independently associated not only with fibrosis stage progression but also with hepatocyte ballooning and lobular inflammation—key histological features of MASH (Seki et al., 2002). Researchers have also found that the concentration of MDA in the peripheral blood of patients with MASLD is significantly higher than that in healthy controls, and the oxidation level of their low-density lipoprotein (LDL) is also significantly increased (Zelber-Sagi et al., 2020). In a cross-sectional study of 50 patients with imaging-confirmed MASLD, elevated MDA was significantly associated with more severe hepatic steatosis (Lugo et al., 2026). Free polyunsaturated fatty acids (PUFAs), as the main substrates, are catalyzed to generate polyunsaturated fatty acid-containing phospholipids (PUFA-PLs) through a series of key enzymes, including members of the long-chain acyl-CoA synthetase family 4 (ACSL4), lysophosphatidylcholine acyltransferase 3 (LPCAT3), and LOXs (Yang et al., 2016; Kagan et al., 2017). Using machine learning in 400 patients, Matboli et al. showed that the ferroptosis signature (ACSL4/LPCAT3 upregulation, GPX4 downregulation) distinguishes MASLD from MASH, identifying peroxidation–antioxidant imbalance as the core driver of disease progression (Matboli et al., 2025). These patient-level data link ferroptosis-related lipid peroxidation to both metabolic dysfunction and fibrotic progression across the MASLD spectrum.

##### 4.2.2.1 *System Xc* ^*–*^ */GSH/GPX4* axis

The antioxidant pathway centered on GPX4 and its upstream regulator Nrf2 represents a major research focus, consistent with the keyword burst detection identifying ‘transcription factor Nrf2’ as the term with the highest burst strength (Figure 8C). The *System Xc* ^*-*^ */GSH/GPX4* axis is the core antioxidant pathway responsible for inhibiting cellular ferroptosis. *System Xc* ^*-*^, a heterodimeric protein widely distributed in the cell membrane, consists of SLC7A11 and SLC3A2 subunits. It mediates the coupled process of extracellular cystine uptake and intracellular glutamate efflux through a 1:1 antiport mode, serving as a crucial molecular basis for safeguarding cellular antioxidant function. Extracellular cystine internalized by cells can be converted into cysteine via either the glutathione-dependent pathway or the thioredoxin-thioredoxin reductase 1 (TrxR1) system. As a critical precursor for GSH synthesis, the content of cysteine directly regulates the synthetic efficiency and steady-state level of GSH (Mandal et al., 2010). GSH is the primary non-enzymatic antioxidant in cells and an essential cofactor for GPX4 (Kuganesan et al., 2021). GPX4 is the only enzyme that can directly reduce lipid peroxides within cell membranes, an activity critically dependent on GSH. This reduction halts the lipid peroxidation chain reaction, which maintains membrane integrity and inhibits ferroptosis (Seiler et al., 2008). Consequently, impairing intracellular cysteine availability directly impacts GPX4 activity via GSH, resulting in reduced activity of multiple antioxidant enzymes and dysregulated clearance of lipid peroxides, thereby enhancing cellular sensitivity to ferroptosis.

Emerging evidence indicates that SLC7A11 upregulation protects against inflammation and fibrosis during NASH progression by modulating oxidative stress and energy metabolism (Lv et al., 2024). Arbutin, a natural antioxidant, alleviates MASLD by inhibiting fat mass and obesity-related protein (FTO), which regulates *SLC7A11* methylation and suppresses ferroptosis (Jiang T. et al., 2023). Zhu et al. demonstrated that Thymosin beta 4 (Tβ4) inhibits hepatocyte ferroptosis during the progression of MASLD by upregulating GPX4 expression (Zhu Z. et al., 2021).

##### 4.2.2.2 Signaling pathway dysregulation: The other key modulators

Beyond GPX4, ACSL4 is also critically involved in ferroptosis during the pathogenesis of MASLD and MASH (Wang H. et al., 2024; Duan et al., 2022). ACSL4 activates long-chain PUFAs and integrates them into membrane phospholipids to form oxidant-prone PUFA-PLs. This compositional change impairs membrane antioxidant stability, rendering it highly susceptible to iron overload-induced ROS and triggering lipid peroxidation cascades (Sha et al., 2021). Moreover, ACSL4 synergizes with LPCAT3 to accelerate the resynthesis and membrane integration of PUFA-containing phospholipids, thereby exacerbating membrane lipid peroxidation risk (Wang J. et al., 2024).

Gut microbiota dysbiosis impairs the intestinal mucosal barrier, elevates intestinal permeability and microbial metabolite levels, and promotes the translocation of endotoxins and inflammatory factors to the liver via the gut-liver axis, ultimately contributing to MASLD (Ji et al., 2023; Bian et al., 2025). By modulating gut microbiota homeostasis, enhancing hepatic antioxidant capacity, and suppressing hepatocyte ferroptosis, *Lactobacillus plantarum* 1-two to three exerts a protective effect against the progression of MASLD (Zhang M. et al., 2025). Aberrant gut microbiota metabolites in MASLD significantly increase hepatic iron levels, upregulate TFR and ACSL4 expression, and consequently heighten hepatocyte susceptibility to ferroptosis (Liu et al., 2022).

Moreover, Nrf2 plays a pivotal role in orchestrating antioxidant responses and mitigating ferroptosis. Studies have demonstrated that ginsenoside Rd exerts protective effects against liver injury and steatosis in MASLD via Nrf2 pathway-mediated antioxidant activity and ferroptosis inhibition (Liu W. et al., 2025). Dehydroabietic acid activates the NRF2 antioxidant pathway via binding to Keap1, upregulates the expression of downstream targets including heme oxygenase-1 (HO-1), GSH, and GPX4, and ultimately and ultimately inhibits hepatocyte ferroptosis (Gao et al., 2021). Studies have demonstrated that *Tricholoma mongolicum* polysaccharide (TMP) ameliorates MASLD via a gut-liver bidirectional regulatory mechanism. Specifically, TMP not only reshapes the gut microbiota structure, but also modulates hepatic iron metabolic homeostasis by upregulating Nrf2/GPX4 pathway and FPN1, as well as TFR1, thereby alleviating oxidative stress injury and ferroptosis in hepatocytes (Ma et al., 2025).

### 4.3 Research trends and hotspot directions

As shown in the citation network (Figure 7) and the top 10 highly cited publications (Table 1), the most influential works in this field converge on two interrelated themes: the pathogenic mechanisms of ferroptosis in MASLD and its targeted therapeutic strategies. In-depth analysis of the top ten most cited publications reveals that high-impact research in this field primarily focuses on the pathogenic mechanisms of ferroptosis in MASLD and its targeted prevention and treatment strategies.

Regarding mechanisms, ferroptosis has been identified as a key early driver event in NASH, acting as a “switch” for initiating inflammation. Occurring prior to apoptosis in the early stages of the disease, ferroptosis mediates hepatocyte death through lipid peroxidation, releasing damage-associated molecular patterns (DAMPs) that drive inflammatory responses (Tsurusaki et al., 2019b). This process is tightly regulated by the ATF4-SLC7A11-GPX4 axis. Under normal conditions, ATF4 induces SLC7A11 expression to promote cystine uptake and glutathione synthesis, which sustains GPX4 activity for eliminating lipid peroxides. Conversely, disruption of this axis causes glutathione depletion and renders hepatocytes susceptible to ferroptosis, thereby accelerating the progression from NASH to hepatocellular carcinoma (He et al., 2023; Qi et al., 2020). At the level of specific effector pathways, GPX4 inactivation activates the 12/15-LOX/AIF axis, promoting peroxidation of polyunsaturated fatty acids and ultimately leading to cell membrane rupture (Qi et al., 2020; Wu et al., 2021). Additionally, dysregulated iron metabolism constitutes another core driver of ferroptosis. On one hand, increased uptake of non-transferrin-bound iron via SLC39A14 amplifies oxidative stress through the Fenton reaction. On the other hand, NCOA4-mediated ferritinophagy releases free iron from intracellular stores, creating a vicious cycle that further fuels iron overload and lipid peroxidation (Chen et al., 2022). Notably, the role of ferroptosis exhibits cell-type specificity: in hepatocytes, it promotes inflammation and fibrosis; in hepatic stellate cells, inducing their death exerts anti-fibrotic effects; whereas its role in macrophages remains unclear (Gautheron et al., 2020; Horn and Tacke, 2024). Finally, ferroptosis coexists and interacts with other forms of cell death such as apoptosis, necroptosis, and pyroptosis. Inhibiting a single pathway may activate compensatory death pathways, adding complexity to therapeutic interventions (Gautheron et al., 2020; Shojaie et al., 2020).

Based on the aforementioned regulatory mechanisms, prevention and treatment strategies are primarily developed at the following levels: Firstly, ferroptosis can be directly inhibited through two distinct strategies: iron chelators sequester free iron to block Fenton chemistry, and lipid peroxide scavengers neutralize lipid radicals. Both approaches suppress hepatocyte death and curb the initiation of inflammation at its source (Tsurusaki et al., 2019b; Qi et al., 2020). Secondly, the cellular intrinsic defense system can be enhanced through two complementary routes: GPX4 activators directly boost core antioxidant enzyme activity, and activation of the ATF4-SLC7A11 axis promotes cystine uptake and glutathione synthesis. Both routes restore cellular antioxidant capacity and interrupt the chain reaction of lipid peroxidation (He et al., 2023; Qi et al., 2020). Thirdly, membrane remodeling can be achieved either by ACSL4 inhibitors that limit PUFA incorporation into phospholipids, or by 12/15-LOX inhibitors that abrogate enzymatic lipid peroxidation—both of which diminish ferroptosis susceptibility (Chen et al., 2022; Qi et al., 2020). Lastly, cell type-specific interventions can be implemented by leveraging the opposing roles of ferroptosis in different hepatic cell types via targeted delivery systems. The goal is twofold: to inhibit ferroptosis in hepatocytes for alleviating inflammation and injury, and to selectively induce ferroptosis in hepatic stellate cells for eliminating activated cells and reversing fibrosis (Gautheron et al., 2020; Horn and Tacke, 2024). Although these strategies demonstrate potential in preclinical models, clinical translation still faces critical challenges including optimization of pharmacokinetics, cell-specific delivery, biomarker development, and timing of intervention (Chen et al., 2022; Wu et al., 2021).

From the keyword co-occurrence network, we can identify that high-frequency terms such as “oxidative stress”, “cell death”, “iron overload”, “lipid peroxidation”, and “nrf2” are not only extensively studied in recent years but also closely associated with the pathogenesis of ferroptosis in MASLD. Simultaneously, the visual clustering analysis reveals that clusters including #0 (‘iron overload’), #2 (‘iron homeostasis’), #11 (‘lipid peroxidation’) all converge on key mechanistic drivers of ferroptosis. Furthermore, keyword burst detection shows that mechanism-related terms such as “transcription factor nrf2” and “iron metabolism” have exhibited a sustained burst period. These findings collectively indicate that investigating the pathogenic mechanisms of ferroptosis in MASLD has become a research hotspot in recent years. In-depth exploration of these mechanisms provides critical insights into MASLD pathogenesis and informs strategic directions for diagnosis and treatment. These mechanistic understandings hold promise for driving transformative advances in MASLD therapeutics.

Consistent with these bibliometric trends, clinical studies have increasingly investigated ferroptosis-related biomarkers in MASLD patients. Elevated serum ferritin and hepatic iron deposition correlate with fibrosis stage and long-term prognosis; increased MDA and 4-HNE levels are associated with hepatocyte ballooning and lobular inflammation; and emerging evidence from biopsy transcriptomics and exhaled breath analysis has identified GPX4-reduced ferroptosis signatures and volatile lipid peroxidation products as potential non-invasive markers (Matsuoka et al., 2025; Peleman et al., 2024). Collectively, these clinical observations corroborate the keyword clusters #0 (‘iron overload’) and #11 (‘lipid peroxidation’) and highlight patient-level biomarker research as a translational Frontier bridging mechanistic insights with clinical practice.

### 4.4 Therapeutic strategies

As revealed by our bibliometric characterization (Section 3.7), pharmacological interventions constitute a major research hotspot in this field, with the Nrf2-GPX4 axis being the most frequently targeted pathway—a finding that aligns with its prominent keyword burst (Figure 8C). Since the conceptualization of ferroptosis, research on treating MASLD by modulating ferroptosis has continued to grow. Natural phytochemicals, traditional Chinese medicines, herbal formulas, synthetic compounds, Western medicines, and drug-loaded nanoparticles exert therapeutic effects on MASLD by regulating ferroptosis-related genes and signaling pathways. These interventions exert therapeutic effects in MASLD by regulating iron metabolism, inhibiting lipid peroxidation, enhancing antioxidant systems, and alleviating inflammation and fibrosis. Meanwhile, certain agents also regulate ferroptosis through mechanisms such as modulating gut microbiota (Ning et al., 2020; Gu S. M. et al., 2025) and promoting demethylation (Jiang T. Y. et al., 2023), thereby conferring therapeutic benefits for MASLD (Table 2; Table 3).

## 5 Limitations

This study has several limitations that should be considered when interpreting the findings. Firstly, the analysis relied exclusively on literature indexed in the WOSCC, PubMed and Scopus. Although these databases is a widely used and reputable source for bibliometric studies, relevant publications from other databases such as Google Scholar was not included, which may affect the completeness of the dataset. Secondly, only English-language publications were analyzed, potentially excluding significant research published in other languages. Thirdly, citation-based metrics are influenced by time, as older articles generally accumulate more citations; thus, recently published high-quality work may not yet be fully reflected in citation counts. Furthermore, differences in the algorithms and functional capabilities of the bibliometric tools used could introduce minor variations in the network visualizations and analytical outputs. Finally, the relatively limited sample size (373 publications) and the concentration of publications in the 2023–2025 period represent inherent constraints of analyzing an emerging research field. While CiteSpace’s burst detection algorithm is methodologically suited for identifying short-term frequency surges, the findings from temporal analyses should be interpreted as indicative of recent research hotspots rather than definitive evidence of long-term evolutionary patterns. The burst periods identified reflect the current momentum in specific mechanistic and therapeutic directions, but their sustainability and long-term significance remain to be validated as the field matures. Future bibliometric updates with extended time windows will be valuable for confirming or refining the trends identified in this study. Notwithstanding these constraints, this study provides a systematic overview of the current research landscape on ferroptosis in MASLD, identifies key thematic clusters and evolving trends, and offers a foundational reference for guiding future investigations in this rapidly developing field.

## 6 Conclusion

This research provides a systematic bibliometric analysis of research trends and emerging foci in the field of ferroptosis in MASLD over the past 8 years. The findings reveal a consistent growth in both annual publications and citation frequency, with China and its affiliated institutions and authors contributing prominently to the literature. The analysis underscores the significant role of ferroptosis in MASLD pathogenesis, involving mechanisms such as iron overload, lipid peroxidation, dysregulation of antioxidant systems, and inflammatory-immune responses. Consequently, investigations targeting ferroptosis mechanisms have become a central research focus in recent years. Furthermore, modulating ferroptosis pathways, whether via traditional Chinese medicine or modern biomedical approaches, represents a significant direction for preventing and treating MASLD. Nevertheless, current research remains largely confined to preclinical models, including animal and cellular studies. Future efforts should prioritize deeper mechanistic exploration and clinical translation to bridge the gap between experimental findings and therapeutic applications. These advances are expected to provide novel perspectives and strategies for targeting ferroptosis in the management of MASLD.

## Data Availability

The original contributions presented in the study are included in the article/Supplementary Material, further inquiries can be directed to the corresponding authors.
