# Supplementary material for: Bibliometric analysis and visualization of ferroptosis in metabolic dysfunction-associated steatotic liver disease (MASLD): a comprehensive review
Source: Front Pharmacol. 2026 Aug 17;17:1856946. doi: 10.3389/fphar.2026.1856946 (PMC13526599; doi:10.3389/fphar.2026.1856946)
Supplement: Supplementary file 1 [file Supplementaryfile1.docx]

**Table S1 Search strategy via Web of Science Core Collection**

| **Step** | **Search strategy** |
| --- | --- |
| **#1** | **TS= (NAFLD OR “Non alcoholic Fatty Liver Disease” OR “Non-alcoholic Fatty Liver Disease” OR “Nonalcoholic Fatty Liver Disease” OR “Nonalcoholic Fatty Liver*” OR NASH OR “Nonalcoholic Steatohepatitis” OR “Nonalcoholic Steatohepatitides” OR MAFLD OR “Metabolic Associated Fatty Liver Disease” OR MASLD OR “Metabolic Dysfunction-Associated Steatotic Liver Disease” OR MASH OR “Metabolic Dysfunction-Associated Steatohepatitis”)** |
| **#2** | **TS= (****ferroptosis OR ferroptotic OR Oxytosis)** |
| **#3** | **#1 AND #2** |

**Table S2 Search strategy via Scopus**

| **Step** | **Search strategy** |
| --- | --- |
| **#1** | **( ( TITLE-ABS-KEY ( NAFLD ) OR TITLE-ABS-KEY ( "Non alcoholic Fatty Liver Disease" ) OR TITLE-ABS-KEY ( "Non-alcoholic Fatty Liver Disease" ) OR TITLE-ABS-KEY ( "Nonalcoholic Fatty Liver*" ) OR TITLE-ABS-KEY ( NASH ) OR TITLE-ABS-KEY ( "Nonalcoholic Steatohepatitis" ) OR TITLE-ABS-KEY ( "Nonalcoholic Steatohepatitides" ) OR TITLE-ABS-KEY ( MAFLD ) OR TITLE-ABS-KEY ( "Metabolic Associated Fatty Liver Disease" ) OR TITLE-ABS-KEY ( MASLD ) OR TITLE-ABS-KEY ( "Metabolic Dysfunction-Associated Steatotic Liver Disease" ) OR TITLE-ABS-KEY ( MASH ) OR TITLE-ABS-KEY ( "Metabolic Dysfunction-Associated Steatohepatitis" ) ) ) AND ( ( TITLE-ABS-KEY ( ferroptosis ) OR TITLE-ABS-KEY ( ferroptotic ) OR TITLE-ABS-KEY ( Oxytosis ) ) )** |

**Table S3 Search strategy via PubMed**

| **Step** | **Search strategy** |
| --- | --- |
| **#1** | **"non alcoholic fatty liver disease"[MeSH Terms] OR "NAFLD"[Title/Abstract] OR "non alcoholic fatty liver disease"[Title/Abstract] OR "nonalcoholic fatty liver disease"[Title/Abstract] OR "nonalcoholic fatty liver*"[Title/Abstract] OR "NASH"[Title/Abstract] OR "nonalcoholic steatohepatitis"[Title/Abstract] OR "MAFLD"[Title/Abstract] OR "metabolic associated fatty liver disease"[Title/Abstract] OR "MASLD"[Title/Abstract] OR "metabolic dysfunction associated steatotic liver disease"[Title/Abstract] OR "MASH"[Title/Abstract] OR "metabolic dysfunction associated steatohepatitis"[Title/Abstract]** |
| **#2** | **"ferroptosis"[MeSH Terms] OR "ferroptotic"[Title/Abstract****] OR "****Oxytosis"[Title/Abstract]** |
| **#3** | **#1 AND #2** |
